# Amendment to Somatic mutation of the cohesin complex subunit confers therapeutic vulnerabilities in cancer

**DOI:** 10.1172/JCI207666

**Published:** 2026-05-01

**Authors:** Yunhua Liu, Hanchen Xu, Kevin Van der Jeught, Yujing Li, Sheng Liu, Lu Zhang, Yuanzhang Fang, Xinna Zhang, Milan Radovich, Bryan P. Schneider, Xiaoming He, Cheng Huang, Chi Zhang, Jun Wan, Guang Ji, Xiongbin Lu

Original citation: *J Clin Invest*. 2018;128(7):2951–2965. https://doi.org/10.1172/JCI98727

Citation for this amendment: *J Clin Invest*. 2026;136(9):e207666. https://doi.org/10.1172/JCI207666

The *JCI* previously issued a retraction for this article following joint notification by Indiana University, The Ohio State University, and the University of Maryland, College Park of data falsification in Supplemental Figure 8A and Supplemental Figure 9E and misconduct findings against Xiongbin Lu and Yunhua Liu.

An independent investigation by Longhua Hospital, Shanghai University of Traditional Chinese Medicine has now been completed. Longhua Hospital’s investigative panel determined that there was an error in the use of images due to operational negligence; however, the panel found no evidence of data fabrication, falsification, or other forms of academic misconduct.

This article remains retracted.

## Version 1 05/01/2026

Electronic publication

